# Effect of Statin Use on the Clinical Manifestations, Laboratory Test Results and Outcome of Lyme Neuroborreliosis

**DOI:** 10.3390/jcm9092995

**Published:** 2020-09-16

**Authors:** Katarina Ogrinc, Andrej Kastrin, Stanka Lotrič-Furlan, Petra Bogovič, Tereza Rojko, Tjaša Cerar-Kišek, Eva Ružić-Sabljić, Gary P. Wormser, Franc Strle

**Affiliations:** 1 Department of Infectious Diseases, University Medical Center Ljubljana, 1000 Ljubljana, Slovenia; stanka.lotric-furlan@mf.uni-lj.si (S.L.-F.); petra.bogovic@kclj.si (P.B.); tereza.rojko@kclj.si (T.R.); franc.strle@kclj.si (F.S.); 2 Institute for Biostatistics and Medical Informatics, Faculty of Medicine, University of Ljubljana, 1000 Ljubljana, Slovenia; andrej.kastrin@mf.uni-lj.si; 3 Institute for Microbiology and Immunology, Faculty of Medicine, University of Ljubljana, 1000 Ljubljana, Slovenia; tjasa.cerar@mf.uni-lj.si (T.C.-K.); eva.ruzic-sabljic@mf.uni-lj.si (E.R.-S.); 4 Division of Infectious Diseases, New York Medical College, Valhalla, NY 10595, USA; GWormser@nymc.edu

**Keywords:** Lyme neuroborrelisis, Bannwarth’s syndrome, statin, course, outcome

## Abstract

Statins have anti-inflammatory and potentially antimicrobial activity, but whether they have a beneficial effect on the course of infectious diseases is controversial. In this study, we assessed the impact of pre-existing statin use on the course and outcome of Lyme neuroborreliosis manifested as meningoradiculitis (Bannwarth’s syndrome). One hundred and twenty three consecutive patients with Bannwarth’s syndrome, of whom 18 (14.6%) were being treated with statins, were included in the study. To assess the influence of statin use on the course and outcome of the disease, univariate and multivariable analyses were performed. No statistically significant association was found between statin pre-treatment and the clinical manifestations, laboratory test results, and outcome of Bannwarth’s syndrome. In conclusion, pre-existing use of statins did not significantly impact either the clinical presentation or the outcome of Bannwarth’s syndrome.

## 1. Introduction

Bannwarth’s syndrome is a common and well-established manifestation of Lyme neuroborreliosis (LNB) in Europe. This manifestation is mainly caused by *Borrelia garinii*, which is transmitted to humans by the bite of an *Ixodes ricinus* tick. Bannwarth’s syndrome is characterized by painful radiculoneuritis and lymphocytic pleocytosis in the cerebrospinal fluid (CSF) [1,2]. In about one-third of patients, it is associated with cranial nerve involvement (usually peripheral facial palsy (PFP)), that typically appears days to weeks after the development of radicular pain; more rarely, peripheral paresis may develop [3]. The borrelial etiology is supported by the presence of erythema migrans (EM), which precedes or accompanies the condition in >50% of patients in some series [3], and is proven by a demonstration of intrathecal synthesis of borrelial antibodies and/or by demonstration of *Borrelia burgdorferi* sensu lato in CSF by culture or molecular testing. The outcome after a 14-day course of antibiotic therapy with intravenous ceftriaxone or oral doxycycline is favorable in the large majority of patients [3].

Statins are cholesterol-lowering drugs and are widely used for the prevention of cardiovascular disease. Because of their anti-inflammatory, as well as their antimicrobial activity [4,5,6] along with additional evidence that statins may reduce neuro-inflammation and blood-brain barrier dysfunction [7,8,9], numerous clinical studies have assessed their impact on the outcome of many different infectious diseases [10,11,12,13,14,15,16,17]. However, although statins were reported to have beneficial effects in a murine model of Lyme borreliosis [18], we were unable to find any report on the impact of statins on the severity or course of Lyme borreliosis in humans.

The goal of our study was to evaluate the effect of pre-existing statin use on the course and outcome of LNB manifested as Bannwarth’s syndrome.

## 2. Patients and Methods

### 2.1. Patients

Consecutive patients aged ≥18 years with a clinical diagnosis of Bannwarth’s syndrome (defined by the presence of radicular pain and CSF lymphocytic pleocytosis as a manifestation of LNB) attending our Lyme borreliosis outpatient clinic between November 2005 and December 2017 were included in the study. Demographic, epidemiologic, and clinical data were obtained prospectively using a structured questionnaire. Patients were asked about symptoms that had newly developed or intensified since the beginning of the current illness and had no other obvious explanation. The overall intensity of their subjective symptoms was graded from 0 to 2 (subjective symptoms score): 0—no symptoms, 1—mild to moderate symptoms, 2—severe symptoms (demanding substantial amount of analgesics and/or significantly interfering with their daily activities).

In clinical examination, particular attention was paid to whether EM was present and, if so, its diameter and appearance (diffuse or ring-like), and to the signs of neurological involvement (meningeal signs, PFP, or other cranial nerve involvement, paresis, tremor).

After antibiotic treatment, patients were followed up at outpatient visits at 14 days, and at 3, 6, and 12 months; at each visit the patients’ symptoms and signs were recorded and the subjective symptoms score was graded in the same manner, as at the first visit. A repeat lumbar puncture was performed at the 3-month follow-up visit. 

The clinical presentation and outcome of 77 of these patients, i.e., those diagnosed with Bannwarth’s syndrome between November 2005 and October 2013, have been reported previously [3].

The study was conducted in accordance with The Code of Ethics of the World Medical Association (Declaration of Helsinki) and was approved by the Medical Ethics Committee of the Ministry of Health of the Republic of Slovenia (35/08/06). Each subject provided written consent for the lumbar puncture.

### 2.2. Methods

#### 2.2.1. Laboratory Evaluation

Routine blood analyses (C-reactive protein, blood cell counts, liver and renal function tests) were performed. CSF was tested for leukocyte cell counts and levels of protein and glucose; a leukocyte count >5 × 10^6^ cells/L (pleocytosis) and a protein concentration >0.45 g/L were considered to be abnormal findings. Immunoglobulin classes G and M (IgG and IgM, respectively) and albumin levels were determined in CSF and serum, and the corresponding quotients (CSF/serum) were calculated. Quotients of IgG and albumin were interpreted as abnormal when they were above 0.0035 and 0.0074, respectively.

#### 2.2.2. Serological Evaluation

Up to 2010, antibodies to *B. burgdorferi* sensu lato in serum and CSF were determined by an indirect immunofluorescence assay (IFA) without absorption [19]. A local isolate of *B. afzelii* was used as an antigen. Reactivity at serum dilutions of 1:256 or higher was interpreted as positive, based on the results of a control group from the same geographic region [20]. Since 2010, an indirect chemiluminescence immunoassay (LIAISON^®^, Diasorin, Italy) with recombinant antigens OspC and VlsE for detection of IgM antibody, and VlsE for IgG were used instead. Results were interpreted according to the manufacturer’s instructions. Intrathecal synthesis of borrelial antibodies was determined as described by Reiber and Peter: antibody index values (AI) >2.0 (IFA) or >1.4 (Liaison^®^, Alpharetta, GA, USA) were interpreted as indicating intrathecal production of borrelial antibodies [21].

In the large majority of patients, serum IgM and IgG antibodies to tick-borne encephalitis (TBE) virus were also determined, using Enzygnost Anti-TBEV (IgM, IgG) kits (Dade Behring Marburg GmbH, Marburg, Germany), according to the manufacturer’s instructions.

#### 2.2.3. Cultivation and Typing of *B. burgdorferi* sensu lato

Modified Kelly-Pettenkofer (MKP) medium was used for cultivation of *B. burgdorferi* sensu lato from blood, CSF, and skin specimens as described elsewhere [22]. CSF (1 mL) samples were obtained from all patients; blood (9 mL) samples were obtained from 114 of 123 patients. Skin biopsies were taken from patients who had EM in the course of the disease and consented to the procedure; the sample (2.5 × 2 × 2 mm) obtained with classical skin biopsy, or a 3 mm punch biopsy, was taken at the border of the EM or at the site of past EM (when EM had already disappeared). CSF and skin samples were inoculated directly into tubes containing 7 mL MKP medium. Blood samples were centrifuged, and 1-mL samples of plasma were inoculated into tubes containing 7 mL MKP medium. All samples were cultivated at 33 °C and examined weekly by dark-field microscopy for the presence of spirochetes (up to 9 weeks for skin and CSF samples, 12 weeks for blood). Isolates were identified into species/strain levels using pulsed-field gel electrophoresis after *MluI* restriction of genomic DNA or by polymerase chain reaction–based restriction fragment-length polymorphism of the intergenic region [23,24].

### 2.3. Treatment

Patients were treated for 2 weeks with ceftriaxone 2 g daily intravenously or with oral doxycycline 100 mg twice daily.

### 2.4. Definition of Outcome

The outcome was defined as follows:

- Complete recovery: complete resolution of symptoms/signs of the disease.
- Pronounced improvement: symptoms/signs of the disease are present only occasionally and/or have low intensity, not demanding regular use of analgesics and not interfering with daily activities.
- Partial improvement: symptoms/signs, although improved, still require frequent use of analgesics and/or significantly reduce daily activities.
- Failure: persistence, intensification, or appearance of new symptoms/signs, and/or a positive post-treatment CSF, skin, or blood culture for borrelia.
- Unfavorable long-term clinical outcome was defined as partial improvement or failure at 6 or 12 months after enrollment; all other patients were classified as having a favorable outcome.

### 2.5. Retreatment

Patients were retreated because of unsatisfactory clinical response to the first antibiotic treatment (i.e., patients with symptoms/signs fulfilling criteria for partial response or failure), persistence of CSF pleocytosis (>10 × 10^6^ cells/L), and/or a positive post-treatment CSF, blood, or skin culture for borrelia, 3 or 6 months after the initial treatment. A 2-week course of ceftriaxone or doxycycline was used, as described above.

### 2.6. Statistical Analysis

Continuous variables were summarized with medians and interquartile ranges, while categorical variables were summarized as frequencies and percentages. All percentage values were reported with 95% confidence intervals (CIs).

For the assessment of the association of statin use with acute illness, we used univariate logistic regression and simple linear regression as appropriate. Multiple regression was used to adjust for the differences between statin users and non-users for seven other covariates including age, male sex, underlying illnesses, albumin quotient, IgG quotient, erythema migrans in the course of illness, and antibiotic treatment prior to the visit at our institution. The covariates included in the multivariate model were selected by expert opinion (K.O. and F.S.) and were decided upon without knowledge of the observed outcome.

A similar approach was used to assess the association of statin usage and long-term outcome. Multivariate analysis included statins and 11 other covariates including age, male sex, underlying illnesses, duration of neurological symptoms/signs, muscle paresis, cranial nerve impairment, CSF leukocyte count, CSF protein concentration, albumin quotient, IgG quotient, and intrathecal synthesis of borrelia IgG antibodies (IA index). Where appropriate, Firth logistic regression was employed to reduce the bias of the parameter estimates due to a high number of covariates.

Regression models were summarized with raw and adjusted odds ratios (ORs) for logistic models, and with estimated coefficients (ECs) for linear regression. We also reported 95% confidence intervals and appropriate P values based on the Wald test. Statin non-users were defined as the reference group in all of the statistical models. OR < 1 means that we estimated a lower outcome likelihood for statin users in comparison with non-users. The corresponding meaning for EC value was EC < 0.

To determine the significance of the regression coefficients, we used the P values on each (multiple) regression performed. The Benjamini and Hochberg procedure for correcting the false positive due to multiple comparisons was used to adjust the P values for the regressions performed. All statistical analyses were performed using R software.

## 3. Results

One hundred and twenty-three adult patients with Bannwarth’s syndrome (71 males (57.7%) and 52 females (42.3%), with a median age of 58 (IQR 50–67) years) were included in the study. Eighteen (14.6%) of them were being treated with statins. Two patients had concomitant TBE (neither had received statins). In 110/123 (89.4%) patients, the diagnosis of LNB was established by demonstration of intrathecal antibody synthesis of borrelia antibodies and/or isolation of borrelia from CSF, while in 13 patients (10.6%), the presence of EM suggested the borrelial etiology. The detailed demographic, clinical, and laboratory data of included patients are shown in Table 1.

Patients in the statin group were older and more often had underlying illnesses than in the non-statin group, but the differences were not significant. Radicular pain (100%) and sleep disturbances (79.7%) were the most frequent symptoms. Among clinical signs at presentation, PFP, EM, meningeal signs, and paresis were observed in 37.4%, 33.3%, 21.1%, and 10.6% of patients, respectively. The frequency of each of these signs was comparable between the two groups of patients. CSF examination revealed lymphocytic pleocytosis in all patients (one of the inclusion criteria), an elevated protein concentration in 86.2% of patients, and a glucose level below one-third of the serum glucose level in 4.9% of patients. There were no significant differences in the CSF cell count and protein and glucose levels between patients who had been receiving statins and those who had not. Serological tests revealed positive borrelial IgG antibodies in serum and CSF in 86.2% and 80.5% of patients, respectively, with intrathecal synthesis of borrelial IgG antibodies in nearly 79% of patients. Borrelia were isolated from CSF, blood, and skin samples in 12.2%, 1.7%, and 40.4% of patients, respectively. *B. garinii* predominated among the CSF and skin isolates (86.7% and 100%, respectively).

The outcome of LNB (clinical and laboratory characteristics) according to whether or not there was pretreatment with statins is shown in Table 2. The subjective symptoms score, an unfavorable outcome and the frequency of neurological signs (cranial nerve involvement and paresis) at follow-up visits, as well as the CSF leukocyte count and protein level at 3 months, were comparable between the groups.

The results of univariate and multivariable analyses of the effect of the use of statins on the acute illness and on the outcome of Bannwarth’s syndrome are shown in Table 3 and Table 4. No significant differences were found between those receiving statins and those who were not.

## 4. Discussion

In recent years, statins, which are used to lower cholesterol levels and to prevent cardiovascular disease, have become the object of increasing interest for their potential benefit in patients with infections because of the anti-inflammatory and antimicrobial actions of this drug class. It has been postulated that due to their anti-inflammatory activity, statins might reduce tissue damage from infections and therefore could potentially improve outcomes. However, the published scientific data on the potential clinical benefit of statins in patients with infections have shown variable results, and we were not able to find any report on the impact of statins on the severity or course of Lyme borreliosis. However, a literature search revealed that (i) *B. burgdorferi*, the principal cause of Lyme borreliosis in North America, has a functional 3-hydroxy-3-methyl-glutaryl-coenzyme A reductase (HMGR EC 1.1.1.88), which is a rate-limiting enzyme of the mevalonate pathway that contributes to components critical for cell wall synthesis; (ii) that statins inhibit *B. burgdorferi* in vitro; and (iii) that statins in a murine model of *B. burgdorferi* infection contribute to a reduction of bacterial burden and an alteration of the murine immune response to favor clearance of the spirochetes [18].

In our study, we evaluated the impact of pretreatment with statins on the course and outcome of early European LNB. We assessed a group of 123 well-defined patients with Bannwarth’s syndrome, a frequent and well-established manifestation of LNB in Europe, of whom 14.6% had been treated with statins. Comparison of basic demographic, clinical, and laboratory characteristics of patients who had been receiving statins and those not receiving statin treatment revealed no substantive differences, with the exception that patients receiving statins were older and more often had underlying illnesses; however, after adjusting for other covariates, even the differences in age and underlying illnesses were not statistically significant.

Many parameters were assessed to define the severity of the acute illness. These included the duration and frequency of certain symptoms and signs, CSF analysis results, as well as serological and culture results. By using univariate and multivariable linear or logistic regression, we did not find any differences in these parameters between those patients who had been pretreated with statins and those who had not (Table 3).

The outcome of patients was also systematically evaluated. Outcome was defined based on the intensity of clinical symptoms (subjective symptoms score) and the presence of neurological signs (cranial nerve palsy, paresis) at 3, 6, and 12 months after antibiotic treatment, CSF examination results and the IgG AI value 3 months after treatment, whether there was an unfavorable outcome at the follow up visits, and the need for antibiotic retreatment within 3 to 6 months after the first course of antibiotic therapy. No statistically significant differences were found between patients receiving and not receiving statins by univariate and multivariable linear or logistic regression analyses (Table 4).

Although in the present study we could not find a correlation between statin therapy and clinical presentation or outcome of Bannwarth’s syndrome, the possibility of an impact of statin usage on the course and outcome of Bannwarth’s syndrome could not be conclusively excluded due to the large CIs, probably due to the relatively small sample size (only 18 patients out of 123 had been treated with statins). Another limitation of the present study is that the effect of statins was investigated only for adult patients with Bannwarth’s syndrome, which is mainly caused by *B. garinii* in Europe, and not for other manifestations of Lyme borreliosis, that are caused by other Borrelia species.

## 5. Conclusions

In spite of in vitro findings and the results of a murine model of Lyme borreliosis suggesting a beneficial effect of statins on the course of *B. burgdorferi* infection, we could not prove a favorable impact of statins on the severity of the acute illness or on the outcome of Bannwarth’s syndrome, a frequent and well-established manifestation of early LNB in Europe.

## Figures and Tables

**Table 1:** Demographic, clinical, and laboratory characteristics of 123 patients with Bannwarth’s syndrome.

Characteristic	Number (%, 95% CI) or Median (IQR)		*p*	*p_adj_*
	Receiving Statins			
	Yes (*n* = 18)	No (*n* = 105)		
Sex: Male	11 (61.1, 38.6–79.7)	56 (53.3, 43.8–62.6)	0.722	>0.999
Age (years) Males Females	64.5 (53.2–70.0) 54.0 (49.0–69.0) 69.0 (67.0–70.0)	57.0 (49.0–65.0) 53.0 (46.0–65.0) 60.0 (53.0–66.0)	0.055 0.402 0.008	0.565 >0.999 0.212
Underlying illnesses	18 (100, 82.4–100)	53 (50.5, 41.1–59.9)	<0.001	0.053
EM ^β^	15 (83.3, 60.8–94.2)	61 (58.1, 48.5–67.1)	0.064	0.565
Fever (>38 °C) ^β^	2 (11.1, 3.1–32.8)	19 (18.1, 11.9–26.5)	0.736	>0.999
Nausea ^β^	5 (27.8, 12.5–50.9)	9 (8.6, 4.6–15.5)	0.033	0.437
Vomiting ^β^	2 (11.1, 3.1–32.8)	4 (3.8, 1.5–9.4)	0.212	>0.999
Vertigo ^β^	4 (22.2, 9.0–45.2)	12 (11.4, 6.7–18.9)	0.251	>0.999
Paresthesia ^β^	7 (38.9, 20.3–61.4)	30 (28.6, 20.8–37.8)	0.546	>0.999
Memory disturbances ^β^	1 (5.6, 1.0–25.8)	13 (12.4, 7.4–20.0)	0.691	>0.999
Concentration disturbances ^β^	1 (5.6, 1.0–25.8)	13 (12.4, 7.4–20.0)	0.691	>0.999
Sleep disturbances ^β^	15 (83.3, 60.8–94.2)	83 (79.0, 70.3–85.7)	>0.999	>0.999
Malaise ^β^	6 (33.3, 16.3–56.3)	32 (30.5, 22.5–39.8)	>0.999	>0.999
Fatigue ^β^	9 (50.0, 29.0–71.0)	38 (36.2, 27.6–45.7)	0.395	>0.999
Radicular pain ^β^	18 (100, 82.4–100)	105 (100, 96.5–100)	>0.999	>0.999
Headache ^β^	6 (33.3, 16.3–56.3)	43 (41.0, 32.0–50.5)	0.727	>0.999
Neck pain ^β^	4 (22.2, 9.0–45.2)	22 (21.0, 14.3–29.7)	>0.999	>0.999
Lower back pain ^β^	3 (16.7, 5.8–39.2)	26 (24.8, 17.5–33.8)	0.560	>0.999
Myalgia ^β^	3 (16.7, 5.8–39.2)	23 (21.9, 15.1–30.7)	0.762	>0.999
Arthralgia ^β^	2 (11.1, 3.1–32.8)	19 (18.1, 11.9–26.5)	0.736	>0.999
Duration of neurological symptoms (days) ^β^	23 (13–46)	30 (14–45)	0.923	>0.999
Concomitant TBE	0 (0, 0–17.6)	2 (1.9, 0.5–6.7)	>0.999	>0.999
Findings at Presentation				
EM	9 (50.0, 29.0–71.0)	32 (30.5, 22.5–39.8)	0.176	>0.999
Ring-like EM	4/9 (44.4, 18.9–73.3)	15/32 (46.9, 30.9–63.6)	>0.999	>0.999
Largest diameter of EM (cm)	33.5 (21.8–43.0)	33.5 (20.0–45.8)	0.886	> 0.999
Lymphocytoma	0 (0, 0–17.6)	1 (1.0, 0.2–5.2)	>0.999	>0.999
Fever (>38 °C)	0 (0, 0–17.6)	0 (0, 0–3.5)	>0.999	>0.999
Meningeal signs	5 (27.8, 12.5–50.9)	21 (20.0, 13.5–28.6)	0.532	>0.999
Peripheral facial palsy	9 (50.0, 29.0–71.0)	37 (35.2, 26.8–44.7)	0.351	> 0.999
Bilateral facial palsy	3/9 (33.3, 12.1–64.6)	3/37 (8.1, 2.8–21.3)	0.079	0.598
Other cranial nerve palsy	0 (0, 0–17.6)	1 (1.0, 0.2–5.2)	> 0.999	>0.999
Paresis	3 (16.7, 5.8–39.2)	10 (9.5, 5.3–16.6)	0.404	>0.999
Tremor	0 (0, 0–17.6)	3 (2.9, 1.0–8.1)	>0.999	>0.999
CSF Laboratory Findings				
Leukocyte count (×10^6^ cells/L) ^α^	88.0 (30.0–373.0)	138.0 (64.0–293.0)	0.367	>0.999
Protein concentration (g/L) Elevated (>0.45 g/L)	1.07 (0.54–1.92) 14 (77.8, 54.8–91.0)	1.12 (0.62–1.90) 92 (87.6, 80.0–92.6)	0.797 0.274	>0.999 >0.999
Glucose concentration (mmol/L) CSFglu/Sglu <0.33	3.3 (2.9–3.5) 1/17 (5.9, 1.0–27.0)	2.9 (2.5–3.2) 5 (4.8, 2.1–10.7)	0.026 0.999	0.437 >0.999
Albumin quotient *	0.017 (0.008–0.025)	0.015 (0.009–0.025)	0.660	>0.999
IgG quotient *	0.011 (0.004–0.016)	0.010 (0.005–0.025)	0.311	>0.999
Borrelial Serology (Liaison^®^ or IFA) ^γ^				
Positive serum IgM	9 (50.0, 29.0–71.0)	48 (45.7, 36.5–55.2)	>0.999	>0.999
Positive serum IgG	16 (88.9, 67.2–96.9)	90 (85.7, 77.8–91.1)	>0.999	>0.999
Positive CSF IgM	10 (55.6, 33.7–75.4)	63 (60.0, 50.4–68.9)	>0.999	>0.999
Positive CSF IgG	13 (72.2, 49.1–87.5)	86 (81.9, 73.5–88.1)	0.448	>0.999
IT synthesis of borreliaIg M antibodies present	10 (55.6, 33.7–75.4)	60 (57.1, 47.6–66.2)	>0.999	>0.999
IT synthesis of borrelia IgG antibodies present	12 (66.7, 43.7–83.7)	85 (81.0, 72.4–87.3)	0.565	>0.999
IT synthesis of borrelia IgM or IgG antibodies present	13 (72.2, 49.1–87.5)	87 (82.9, 74.5–88.9)	0.351	>0.999
IgG AI value ^#^	8.1 (3.5–42.5)	19.6 (6.0–41.3)	0.228	>0.999
*Borrelia* Culture Results				
Positive CSF culture	1 ^€^ (5.6, 1.0–25.8)	14 ^δ^ (13.3, 8.1–21.1)	0.695	>0.999
Positive blood culture	0/16 (0, 0–19.4)	2 ^§^/98 (2.0, 0.6–7.1)	>0.999	>0.999
Positive skin culture	4 ^&,$^/12 (33.3, 13.8–60.9)	17 ^¥,$^/40 (42.5, 28.5–57.8)	0.741	>0.999

Abbreviations: CI, confidence interval; EM, erythema migrans; TBE, tick-borne encephalitis; CSF, cerebrospinal fluid; CSFglu/Sglu, ratio between CSF glucose concentration and serum glucose concentration; IFA, immunofluorescence assay; AI, antibody index. ^β^ In the course of the illness up to enrollment. ^α^ In case of a traumatic lumbar puncture, the white cell count was adjusted: for every 1000 × 10^6^/L red cells, 1 × 10^6^/L white cells were subtracted. * Albumin (IgG) quotient is a quotient between CSF and serum albumin (IgG) concentration. ^γ^ Only Liaison was performed in 62 patients, only IFA in 18 patients, both tests in 43 patients. ^#^ Index value of intrathecal synthesis of borrelial IgG antibodies only for patients in whom serological results were obtained by Liaison and in whom AI value >1.4 (11 patients in the statin group, 66 patients in the non-statin group). ^€^ CSF culture was positive in 1 of 17 (5.9%) patients who had received no antibiotic therapy before admission, but not in a patient who had been treated with azithromycin before CSF examination (*p* = 0.944). The isolate was identified as *B. garinii*. ^δ^ CSF cultures were positive in 13 of 84 (15.5%) patients who had not received antibiotics and in 1 of 21 (4.8%) patients who had been treated with antibiotics before CSF examination (*p* = 0.178). There were 12/14 isolates which were identified as *B. garinii* and 2/14 as *B. afzelii*. ^§^ Blood cultures were positive in 2 of 77 (2.6%) patients with no previous antibiotic therapy and in 0 of 21 patients who had received antibiotics (*p* = 0.616). Both blood isolates were identified as *B. afzelii*. ^&,^ Borrelia were cultured from the skin samples of 4 of 12 patients without previous antibiotic treatment. ^$^ All skin isolates were identified as *B. garinii*. ^¥^ Skin cultures were positive in 16 of 30 (53.3%) patients who had not been treated with antibiotics, and in 1 of 10 (10%) patients who had received antibiotics before the skin samples were collected (*p* = 0.018).

**Table 2:** The outcome of patients with Bannwarth’s syndrome at certain follow-up visit.

Characteristic	Number (%, 95% CI) or Median (IQR)		*p*	*p*_adj_
	Receiving Statins			
	Yes (*n* = 18)	No (*n* = 105)		
SS score ^¥^ = 0 (3 mo)	11 (61.1, 38.6–79.7)	56/103 (54.4, 44.8–63.7)	0.784	0.968
SS score ^¥^ = 0 (6 mo)	12 (66.7, 43.7–83.7)	52/101 (51.5, 41.9–61.0)	0.350	0.744
SS score ^¥^ = 0 (12 mo)	13 (72.2, 49.1–87.5)	66/100 (66.0, 56.3–74.5)	0.807	0.968
Cranial nerve palsy (3 mo)	4/9 (44.4, 18.9–73.3)	9/35 (25.7, 14.2–42.1)	0.414	0.744
Cranial nerve palsy (6 mo)	3/9 (33.3, 12.1–64.6)	4/34 (11.8, 4.7–26.6)	0.147	0.529
Cranial nerve palsy (12 mo)	3/9 (33.3, 12.1–64.6)	2/33 (6.1, 1.7–19.6)	0.057	0.516
Paresis (3 mo)	2/3 (66.7, 20.8–93.9)	3/10 (30.0, 10.8–60.3)	0.510	0.744
Paresis (6 mo)	1/3 (33.3, 6.1–79.2)	3/9 (33.3, 12.1–64.6)	0.999	0.999
Paresis (12 mo)	0/3 (0.0, 0.00–56.1)	3/9 (33.3, 12.1–64.6)	0.509	0.744
CSF Leukocyte count * (3 mo)	4 (3–8)	6 (2–10)	0.537	0.744
CSF Leukocyte count >10 * (3 mo)	3/16 (18.8, 6.6–43.0)	21/94 (22.3, 15.1–31.8)	0.999	0.999
CSF protein concentration ^$^ (3 mo)	0.49 (0.40–0.58)	0.50 (0.39–0.58)	0.868	0.977
Borrelia IgG AI value ^#^ (3 mo)	11.4 (3.7–25.3)	19.7 (11.5–28.9)	0.140	0.529
Partial improvement or failure (14 days)	10 (55.6, 33.7–75.4)	32/104 (30.8, 22.7–40.2)	0.076	0.516
Partial improvement or failure (3 mo)	4 (22.2, 9.0–45.2)	15/103 (14.6, 9.0–22.6)	0.481	0.744
Partial improvement or failure (6 mo)	3 (16.7, 5.8–39.2)	11/101 (10.9, 6.2–18.5)	0.443	0.744
Partial improvement or failure (12 mo)	4 (22.2, 9.0–45.2)	8/100 (8.0, 4.1–15.0)	0.086	0.516
Antibiotic retreatment (3 or 6 mo)	2 (11.1, 3.1–32.8)	25/103 (24.3, 17.0–33.4)	0.357	0.744

Abbreviations: CI, confidence interval; IQR, interquartile range; SS score, subjective symptoms score; mo, months; CSF, cerebrospinal fluid; AI, antibody index. All 18 patients receiving statins attended a follow-up visit at 3, 6, and 12 months, while in the reference group, 103, 101, and 100 patients attended a corresponding follow up visit. ^¥^ SS score was graded from 0 to 2: 0 (no subjective symptoms), 1 (mild to moderate subjective symptoms), 2 (severe subjective symptoms).* × 10^6^ cells/L. ^$^ g/L. ^#^ Index value of intrathecal synthesis of borrelial IgG antibodies only for patients in whom serological results were obtained by Liaison and in whom AI value >1.4 (13 patients in the statin group, 63 patients in the non-statin group).

**Table 3:** Effect of statin usage on acute illness.

Factors (Covariates)	Est	SE	Stat	*p*	*p*_adj_	CI_L_	CI_H_	Model
Univariate Regression Model								
Cranial nerve involvement	1.84	0.51	1.18	0.236	0.646	0.66	5.10	logistic
Paresis	1.90	0.71	0.90	0.369	0.646	0.39	7.09	logistic
Positive CSF borrelial culture	0.38	1.07	−0.90	0.368	0.646	0.02	2.10	logistic
Duration of neurological symptoms/signs	−0.10	0.25	−0.42	0.678	0.791	−0.60	0.39	linear
CSF leukocyte count	−0.22	0.32	−0.68	0.497	0.696	−0.86	0.42	linear
CSF protein conc	0.00	0.18	0.00	0.999	0.999	−0.37	0.37	linear
Borrelia IgG AI value ^#^	−0.47	0.43	−1.11	0.271	0.646	−1.33	0.38	linear
Multivariate Regression Model								
Cranial nerve involvement	4.06	0.70	2.01	0.044	0.154	1.05	16.61	logistic
Paresis	1.21	0.98	0.19	0.847	0.847	0.14	7.77	logistic
Positive CSF borrelial culture	0.33	1.16	−0.95	0.344	0.602	0.02	2.40	logistic
Duration of neurological symptoms/signs	0.12	0.26	0.47	0.640	0.847	−0.39	0.63	linear
CSF leukocyte count	0.08	0.26	0.31	0.754	0.847	−0.44	0.60	linear
CSF protein conc	0.14	0.06	2.43	0.017	0.119	0.03	0.25	linear
Borrelia IgG AI value ^#^	−0.54	0.51	−1.05	0.297	0.602	−1.57	0.48	linear

Abbreviations: Est, estimate (odds ratio or linear regression coefficient); SE, standard error; Stat, corresponding statistic (*z*-value for logistic and ordinal regression, *t*-value for linear regression); *p*_adj_, adjusted *p*-value using Benjamini and Hochberg correction; CI_L_, lower limit of 95% confidence interval; CI_H_, higher limit of 95% confidence interval; CSF, cerebrospinal fluid; conc, concentration; AI, antibody index. ^#^ Index value of intrathecal synthesis of borrelial IgG antibodies only for patients in whom serological results were obtained by Liaison and in whom AI value >1.4 (11 patients in the statin group, 66 patients in the non-statin group).

**Table 4:** Effect of statin usage on the outcome of Lyme neuroborreliosis.

Factors (Covariates)	Est	SE	Stat	*p*	*p*_adj_	CI_L_	CI_H_	Model
Univariate Regression Model								
SS score * (3 mo)	−0.39	0.51	−0.76	0.447	0.753	−1.43	0.58	ordinal
SS score * (6 mo)	−0.69	0.53	−1.29	0.196	0.417	−1.80	0.32	ordinal
SS score * (12 mo)	−0.16	0.57	−0.28	0.781	0.885	−1.37	0.91	ordinal
Cranial nerve palsy (3 mo)	2.98	0.67	1.64	0.101	0.286	0.73	10.59	logistic
Cranial nerve palsy (6 mo)	4.85	0.81	1.94	0.052	0.269	0.88	24.19	logistic
Cranial nerve palsy (12 mo)	9.80	0.95	2.39	0.017	0.269	1.51	79.23	logistic
Paresis (3 mo)	4.17	0.95	1.50	0.134	0.325	0.52	27.08	logistic
Paresis (6 mo)	1.92	1.18	0.55	0.581	0.760	0.09	16.04	logistic
Paresis (12 mo)	0.75	1.57	−0.18	0.857	0.911	0.03	16.36	logistic
CSF leukocyte count (3 mo)	−0.13	0.24	−0.56	0.577	0.760	−0.60	0.33	linear
CSF leukocyte count (3mo) > 10 × 10^6^/L	0.80	0.69	−0.32	0.748	0.885	0.17	2.78	logistic
CSF protein conc (3 mo)	0.00	0.09	0.00	0.999	0.999	−0.19	0.19	linear
Borrelia IgG AI value ^#^ (3 mo)	−0.47	0.26	−1.84	0.069	0.269	−0.99	0.04	linear
Partial Impro + Failure (2 w)	2.81	0.52	1.99	0.047	0.269	1.02	8.02	logistic
Partial Impro + Failure (3 mo)	1.68	0.63	0.82	0.414	0.753	0.43	5.45	logistic
Partial Impro + Failure (6 mo)	1.64	0.71	0.70	0.487	0.753	0.34	6.01	logistic
Partial Impro + Failure (12 mo)	3.29	0.68	1.76	0.079	0.269	0.79	11.99	logistic
Multivariate Regression Model								
SS score * (3 mo)	0.83	0.77	−0.24	0.808	0.884	0.17	3.61	ordinal
SS score * (6 mo)	0.71	0.80	−0.43	0.666	0.884	0.14	3.26	ordinal
SS score * (12 mo)	0.81	0.87	−0.24	0.814	0.884	0.13	4.19	ordinal
Cranial nerve palsy (3 mo)	0.65	1.21	−0.35	0.723	0.884	0.06	7.02	logistic
Cranial nerve palsy (6 mo)	1.97	1.23	0.55	0.581	0.884	0.18	21.99	logistic
Cranial nerve palsy (12 mo)	13.38	1.41	1.84	0.066	0.561	0.84	212.48	logistic
Paresis (3 mo)	8.58	1.73	1.24	0.214	0.884	0.29	254.94	logistic
Paresis (6 mo)	1.30	1.80	0.15	0.884	0.884	0.04	44.00	logistic
Paresis (12 mo)	1.33	1.79	0.16	0.872	0.884	0.04	44.14	logistic
CSF leukocyte count (3 mo)	1.04	1.48	0.70	0.486	0.884	−1.92	3.99	linear
CSF leukocyte count (3 mo) >10 × 10^6^/L	1.99	1.21	0.57	0.571	0.884	0.18	21.4	logistic
CSF protein conc (3 mo)	0.01	0.06	0.19	0.850	0.884	−0.11	0.14	linear
Borrelia IgG AI value ^#^ (3 mo)	−5.14	6.80	−0.76	0.454	0.884	−18.83	8.56	linear
Partial Impro + Failure (2 w)	1.66	0.76	0.67	0.502	0.884	0.38	7.37	logistic
Partial Impro + Failure (3 mo)	1.51	0.86	0.48	0.631	0.884	0.28	8.21	logistic
Partial Impro + Failure (6 mo)	4.13	1.02	1.40	0.162	0.884	0.56	30.26	logistic
Partial Impro + Failure (12 mo)	15.17	1.20	2.27	0.023	0.391	1.46	157.97	logistic

Abbreviations: Est, estimate (odds ratio or linear regression coefficient); SE, standard error; Stat, corresponding statistic (*z*-value for logistic and ordinal regression, *t*-value for linear regression); *p*_adj_, adjusted *p*-value using Benjamini and Hochberg correction; CI_L_, lower limit of 95% confidence interval; CI_H_, higher limit of 95% confidence interval; SS score, subjective symptoms score; mo, months; CSF, cerebrospinal fluid; conc, concentration; AI, antibody index; impro, improvement; w, weeks. * SS score was graded from 0 to 2: 0 (no subjective symptoms), 1 (mild to moderate subjective symptoms), 2 (severe subjective symptoms). ^#^ Index value of intrathecal synthesis of borrelial IgG antibodies only for patients in whom serological results were obtained by Liaison and in whom AI value >1.4 (13 patients in the statin group, 63 patients in the non-statin group).
